# Cost of non-persistence with oral bisphosphonates in post-menopausal osteoporosis treatment in France

**DOI:** 10.1186/1472-6963-11-151

**Published:** 2011-06-25

**Authors:** François-Emery Cotté, Gérard De Pouvourville

**Affiliations:** 1 CERMES, IFR69, INSERM U750, National Institute of Health and Medical Research, Villejuif, France; 2 Health Outcomes Studies, Laboratoire GlaxoSmithKline, Marly le Roi, France; 3 ESSEC Business School, Chair of Health Economics, Cergy, France

**Keywords:** osteoporosis, bisphosphonate, adherence, persistence, cost, modelling study

## Abstract

**Background:**

During the last decade, oral bisphosphonates (BP) became the most widely prescribed pharmacologic class for post-menopausal osteoporosis. However, many surveys revealed the important issue of poor persistence with those drugs resulting in a failure of treatment to reduce fracture risk sufficiently. Using a published Markov model, this study analyses the economic impact of non-persistence with bisphosphonates in the context of the introduction of generics in France.

**Methods:**

Direct costs of vertebral, hip and wrist fracture were assessed and included in an existing 10-year Markov model developed to analyse consequences of non-persistence. Three alternatives of comparison were set: no treatment, real-world persistence, and ideal persistence. Simulated patients' characteristics matched those from a French observational study and the real-world adherence alternative employed persistence data from published database analysis. The risk of fracture of menopausal women and the risk reduction associated with the drugs were based on results reported in clinical trials. Incremental cost-effectiveness ratios (ICERs) were calculated first between real-world adherence and no treatment alternatives, and second between ideal and real-world persistence alternatives. The cost of non-persistence was defined as the difference between total cost of ideal and real-world persistence alternatives.

**Results:**

Within fractured women population, mean costs of 10-year management of fracture were significantly different between the three alternatives with €7,239 (± €4,783), €6,711 (± €4,410) and €6,134 (± €3,945) in the no-treatment, the real-world and ideal persistence alternatives, respectively (p < 0.0001). Cost-effectiveness ratio for real-world treatment persistence compared with no-treatment alternative was found dominant and as well, alternative of ideal persistence dominated the former. Each ten percentage point of persistence gain amounted to €58 per patient, and extrapolation resulted in a global annual cost of non-persistence of over €30 million to the French health care system, with a substantial transfer from hospital to pharmacy budgets.

**Conclusion:**

Within term, improving persistence with oral bisphosphonates should be economically dominant on levels currently known in real-world. Given this potential savings, ambitious adherence-enhancing interventions should be considered in osteoporotic patients.

## Background

Osteoporosis is characterised by low bone mass and microarchitectural deterioration of bone responsible for 8.9 million fractures each year worldwide [1]. With at least 150,000 clinical fractures every year, preventing osteoporosis within elderly represents an important issue for public health stakeholders in France [2]. This was brought to light in 2007 by reimbursement decisions for osteoporosis screening (i.e. bone mineral densitometry examination) and for primary prevention (i.e. osteoporotic patient without fracture history).

Randomized clinical trials providing evidence for clinical relevant fracture protection by the bisphosphonates [3,4], international guidelines recommended bisphosphonates for the treatment of postmenopausal osteoporosis in the late 1990s [5]. During the following decade, bisphosphonates became the most widely prescribed pharmacologic class for this pathology [6], with nearly 80% of specific drug prescriptions [7] and also corresponding to over €300 million reimbursement by French health system (called Assurance Maladie) for 2008 [8]. Recently, authorities stress the pressure on oral bisphosphonates prices strengthened by near future genericisation of the whole class, as it already occurred in 2008 to the first historical molecule (i.e. alendronate).

Post-menopausal osteoporosis prevalence was estimated at around 30% in women fifty years of age and over [9]. In 2006, a study in French general population showed that this diagnosis was already done to almost 10% of those women highlighting an underdiagnosis issue [10]. At the time of this survey, only 61% claimed receiving an osteoporosis treatment also revealing a significant health management issue with those drugs, in accordance to many other findings [11]. A report of data pooled from the US, UK and France also revealed a one-year discontinuation rate of approximately 50% in women treated with bisphosphonates [12]. Alarmed by low rates of treatment adherence in developed countries for many chronic diseases such as osteoporosis, the World Health Organisation has emphasised the growing body of evidence that supports the notion that increasing the effectiveness of adherence interventions may have a far greater impact on the health of the population than any improvement in the efficacy of specific medical treatments [13]. An expert consensus in osteoporosis defined adherence as a general term encompassing both compliance and persistence [14]. The International Society for Pharmacoeconomic and Outcomes Research (ISPOR) tended to consider terms "adherence" and "compliance" as synonym [15] representing "the extent to which a patient acts in accordance with the prescribed interval and dose of a dosing regimen". Persistence was defined as "the duration of time from initiation to discontinuation of therapy" which corresponds to a continuous variable (i.e. number of days with drug availability) or a dichotomous variable (i.e. being "persistent" or "nonpersistent" at the end of a predefined time period). Graphical illustration of persistence is generally performed according survival analysis following the Kaplan-Meier method so called persistence curve [14].

Although non-persistence was found to systematically reduce treatment effectiveness, this issue was not often taken into account in pharmacoeconomic models [16]. On behalf of the Economics of Medication Compliance Working Group of ISPOR, *Hughes et al. *[17] have formulated methods that may be appropriate for integrating these aspects in economic evaluations. Especially, Markov models were identified to allow direct inclusion of persistence variable in the analysis, as patients transit during each cycle. In 2009, such model was designed to simulate the impact of improving persistence rates on osteoporosis treatment effectiveness [18]. Compared to a non-treatment hypothesis, the relative risk of fracture over ten years was 0.83 for weekly bisphosphonate treatment and decreased to 0.73 if hypothetical full-treatment persistence was achieved. Moreover, improving persistence by 20% was found to have the same clinical impact as a 20% increase in clinical efficacy. In observational studies, women considered as persistent appeared to benefit from a significant decrease of fracture risk ranging from 26% to 32% when compared to nonpersistent ones [19-21]. Nonpersistent patients (i.e. under one-year compliance) were shown to be associated with higher hospitalization rates and higher charges for in-and outpatient health care resources [22]. However, drug expenditures related to non-persistence rates appeared lower [21] and impact of overall incremental direct costs and savings on healthcare budget remains unclear. To our knowledge, no previous study proposed a global approach to estimate cost of non-persistence in the field of osteoporosis.

The modelling study described here proposes an estimation of the budget impact of non-persistence with bisphosphonates, by assessing cost-effectiveness of "ideal persistence" compared to current persistence in real-world and estimating the annual total cost of non-persistence for France.

## Methods

### Model Structure

This modelling study used a validated Markov model that has been developed (TreeAge Software, Williamstown, MA, USA) to predict the incidence of osteoporotic fractures in women with post-menopausal osteoporosis (PMO) in relation with persistence rates with bisphosphonates [18]. The main adaptation is that cost information was linked to the model by assigning each Markov state rewards for costs. These rewards contributed to the total accumulated sum of costs depending on the patient's path through the health states.

Costs and events were firstly estimated for three alternatives: no-treatment, real-world persistence, and ideal persistence. No-treatment alternative modelized the natural history of PMO without assistance of any pharmacologic therapy. The ideal persistence alternative considered bisphosphonates benefits from clinical trials. The real-world persistence alternative assumed all patients were treated by weekly or monthly bisphosphonates and was based on current persistence rates from observational studies. Costs of fractures management and rehabilitation were also added as well as costs of bisphosphonates as per the persistence rate.

The fracture events evaluated were restricted to the hip, the vertebrae and the wrist, since these are the specific sites where the most frequent osteoporotic fractures occur [23] and were particularly well documented compared to less common sites. Morphometric (ie identified by radiography) and symptomatic (*ie *clinically diagnosed) vertebral fractures were differentiated. Input variables for the model included the prevalence of diagnosed PMO, fracture rates at the sites of interest, efficacy and residual beneficial effect of bisphosphonate treatment, and treatment persistence rates. Fracture events were modelled over a ten-year period.

**Figure 1:**
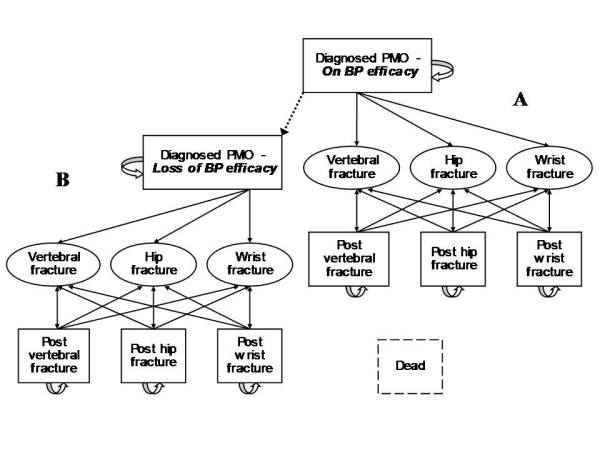
**Markov model structure**.

Key model characteristics were as follows. Markov state transitions consisted in four mutually exclusive health states with a probability of experiencing different fracture events i.e. diagnosed PMO, post-hip fracture, post-vertebral fracture and post-wrist fracture (Figure 1). For every patient and at any 1-year cycle, death could end the simulation. This was developed to simulate the number of osteoporotic hip, vertebral, and wrist fractures as a function of demographic change and other influences. This model was analysed using individual, first-order Monte Carlo simulation, and was pre-designed to be adaptable for assessing the impact of non-persistence improvement on cost.

### Transition probabilities

Transition probabilities between the different health states were defined in the model by features of the French patients diagnosed with PMO in terms of age distribution and proportion of patients with a prior fracture [7]. Annual absolute fracture risks in women with post-menopausal osteoporosis were from estimations based on general population data [24] and relative risk ratios for the association between subsequent and prior fractures from a meta-analysis [25]. Pooled bisphosphonates efficacies for each fracture sites were given by the Health Technology Assessment guidance for health economic models in osteoporosis [26]. Residual effect data from a follow-up of postmenopausal Danish women during 7 years after treatment withdrawal [27] were modelized to determine the relation of treatment duration and treatment global protection i.e. 10-year protection achieved with around 4 years of continued treatment [18].

Insertion of persistence with bisphosphonates treatments occurred in this model only for real-world alternatives. Data were from the Longitudinal Patient Database (LPD) which is constituted by a French representative network of over 1,200 computerized general practitioners (Thales network). Analyses of treatment discontinuation within women newly treated with weekly bisphosphonates showed that 49% of them were nonpersistent after 1-year of follow-up [12] and this rate increased to 59% after 2 years [28]. Persistence curves were extrapolated using the best-fit logarithmic derivations from which functions were combined with those of residual protection following interruption of treatment. Proportion of women with effective protection at each cycle of simulation was then calculated to allow the path of patients from the initial model (Figure 1 - Part A) where they benefit from treatment protection to a twin model (Figure 1 - Part B) where it is not the case anymore. Alternatives as ideal persistence and no-treatment have been individually run in one of the two parts giving extreme values this proportion, respectively one and null.

Baseline age-specific, all-cause mortality rates were derived from 2004 (French national population statistics, INSEE). Mortality following a hip fracture was estimated by adjusting baseline, age-specific, and all-cause mortality using Swedish data [26]. Concerning vertebral fractures, relative risks of mortality in the subsequent year was taken from the European Prospective Osteoporosis Study (EPOS) [29]. This model did not apply incremental mortality to the post-wrist fracture state as neither early nor medium term mortality has been shown [30].

Primary data inputs are summarized in Table 1 including references.

**Table 1:** Model inputs

Clinical inputs for transition probabilities	Values			Sources
***Baseline features***				
Age distribution (mean in years)	70.5			*Blotman et al. 2007 *[7]
Prior fracture (%)	59.7			

***Fracture probabilities***				
*Absolute risk by age (% per year)*	*Vertebrae*	*Hip*	*Wrist*	
50-54 years	3.10	0.00	2.99	
55-59 years	3.59	0.06	3.20	
60-64 years	4.15	0.20	3.18	*Cotté et al. 2009 *[24]
65-69 years	4.81	0.34	2.06	
70-74 years	5.56	0.65	1.97	
75-79 years	6.44	1.04	1.17	
80-84 years	7.45	1.62	0.92	
> 84 years	8.62	3.52	0.92	

*Relative risk*	*Vertebrae*	*Hip*	*Wrist*	
associated with any prior fracture at baseline	2.0	2.0	1.9	
post-vertebral fracture health state	4.4	2.3	1.4	*Klotzbuecher et al. 2000 *[25]
post-hip fracture health state	2.5	2.3	NA	
post-wrist fracture health state	1.7	1.9	3.3	

*Relative risk reduction with BP*	*Vertebrae*	*Hip*	*Wrist*	
for any women at baseline	0.526	0.672	0.833	
from post-vertebral fracture health state	0.575	0.620	NA	*Kanis et al. 2002 *[9]
from post-hip fracture health state	NA	0.620	NA	
from post-wrist fracture health state	0.575	0.620	0.566	

***Loss of bisphosphonate protection***				
Treatment duration (*T*; in months)	*Persistence rates *(*P*; in %)			
Start	100			*Fardellone et al. 2005 *[28]
6	65			*Source data were fitted to an exponential function to generate persistence curve over 24 months:
12	51			*P *= 1 - 0.196 × ln(*T*)
24	41			
36	30*			
48	24*			
60 to simulation ending time	20*			

Treatment duration (*T*; in months)	Protection duration** (y; in months)			
Start	0			*Cotté et al. 2009 *[39]
12	24			**Residual effect of BP was modeled as follow:
24	43			*y *= 13.5 × e^0.048 × *T*^
36	76			
48	All simulation period			
60 to simulation ending time	All simulation period			

### Costs

All costs are given in Euro (€) for the year 2010. An annual discount rate of 5% was used. A sensitivity analysis was also performed without any discounting.

Cost of bisphosphonate treatments were set based on the public price in 2010 extracted from the Caisse Nationale d'Assurance Maladie database [31]. The French authorities have approved generic forms of alendronate 70 mg in 2006 [32], and although risedronate is still protected by a market patent, the base case model assumed by default the generic price of alendronate for bisphosphonates corresponding to €52.23 for 12 weeks of treatment. In a sensitivity analysis, risedronate branded price of €87.46 for 12 weeks was also tested. In the ideal persistence alternative, drug costs were equal to one year of medication at each cycle, whereas in the real-world persistence alternative, those costs were multiplied by persistence rates associated for each cycle.

Considering all vertebral fractures, only 23% were considered to be symptomatic [44] and were assumed to use medical resources. No cost/reward was given to other morphometric-defined vertebral fractures. Because of the lack of accurate data in the literature, cost of symptomatic fractures management in France was estimated. Calculations were based on the medicalization program information system (PMSI) [33] and the National Cost Study (Etude Nationale de Coûts) [34] which are the two independent general sources of information currently available in France about public and private hospital activity and costs. Disease-related groups (DRG) attributed to patients with vertebral fractures as main diagnosis for hospitalizations in 2008 were collected from the PMSI database. National Cost Study which integrates the results of detailed accounting data on a national sample of French hospitals gave cost per each DRG. Those data allowed the calculation of fracture hospitalization cost by weighted average. Although more data are available about non-vertebral fractures, costs of hip and wrist fractures were assessed by the same way for consistency and to validate this method. In PMSI database, two principal ICD 10 diagnostics of hospitalization were used to code both hip fractures (e.g. S720 and S7200) and wrist fracture (e.g. S525 and S526). Vertebral fractures were classified among 21 principal diagnosis (e.g. main prefix: M485, S220, S221, S320, S327 and, S328). Incidences of fractures encoded by principal diagnosis in the PMSI 2007database were as follow: 26,490 at vertebra, 53,376 at hip and 10,394 at wrist. More than 95% of those cases were associated with respectively 12, 8 and 10 different DRGs. For each fracture site, average costs of hospitalisation and global management including rehabilitation [35] were estimated and presented in Table 2.

**Table 2:** Direct costs of osteoporotic fractures management in France (euros 2010)

Fracture site	Hip	Wrist	Vertebra
**Principal diagnosis**			
Codes	S720-S7200	S525-S526	M485(0-9); S22(0-1); S2200; S2210; S320(0); S327(0); S328(0)
Number	53,376	10,394	26,490

Cost of disease-related groups*			
Median cost	€7,170	€2,615	€2,085
Min-Max costs	€1,992 - €15,720	€614 - €3,363	€640 - €15,720

**Direct cost of fracture hospitalizations**			
All fractures	€372,849,923	€18,775,427	€93,325,278
Weighted average cost per unit	€7,308	€1,844	€3,523

Direct cost of management of fracture**			
	**€11,419**	**€3,305**	**€5,872**

*DRGs associated with less than 1% of all cases were not taking into account (PMSI 2008).

**Assumption of 40.0%, 36.0% and 44.2% of rehabilitation cost proportion for vertebral, hip and wrist fractures respectively [35]

A Student t-test was performed to compare mean values of cost with a significance threshold P value of 0.05.

### Analyses

Ten-year period was considered as an appropriate horizon in the field of osteoporosis [36], and the model simulations were also ran over 10 cycles. After stability of the model was checked using predictable random sequences, 30,000 Monte Carlo microsimulations were performed for each alternative, and fractures, costs, and deaths were recorded.

As a primary analysis, incremental cost-effectiveness ratios (ICERs) were calculated first between real-world adherence and no treatment alternatives, and second between ideal and real-world persistence alternatives as described in the following formulas:

Cost criterion (*μ_C_*) encompassed both management of fracture as well as drug consumption, while two effectiveness criteria (*μ_E_*) were assessed: the proportion of fractured women and the proportion of premature deaths at the model horizon. Additional simulations assessed the influence of discounting on the ICERs results.

The cost of non-persistence was defined as the difference between costs balance sheet of ideal and real-world persistence alternatives. Global cost of non-persistence has been estimated by extrapolation of this result to the overall population of diagnosed osteoporotic women estimated at 1.13 million women in France [18]. Cost of management of fracture, drug cost and sum of both, were also studied in function of persistence rate.

## Results

### Simulation outcomes

After 30,000 patients' simulations, the total number of clinical fractures with no treatment alternative achieved 20,701 (Table 3). Real-world treatment persistence and ideal persistence alternatives decreased this total to 16,711 and 12,378 fractures respectively. Considering that several fractures could occurred to a same woman, the number of fractured women was lower than number of fractures in the three alternatives with 14,258 (49.1%), 12,331 (60.0%) and 9,752 (67.1%) respectively. In the no-treatment alternative, where the mean start age was 71.1 ± 9.7 years, 10,080 (33.6%) were died at the end of the 10-year horizon of the model. Among them, 1,816 (18.0%) died following a clinical fracture (1,052 and 764 at hip and vertebral sites, respectively). Compare to this, the excess of mortality were reduce by 350 and 801 deaths in the real-world and ideal persistence alternatives, respectively.

**Table 3:** 10-year Monte-Carlo simulation outcomes

*Monte-Carlo simulations (N = 30,000)*		No-treatment	Real-world persistence	Ideal persistence
**Clinical fractures**	**Vertebrae**	8,193	6,308	3,912
	**Hip**	5,534	4,462	3,313
	**Wrist**	6,674	5,941	5,153
	**Total**	20,401	16,711	12,378

**Fractured women** **(proportion)**		20,131 (67,1%)	17,996 (60.0%)	14,715 (49.1%)

**Deaths** **(proportion)**		1,816 (6.1%)	1,466 (4.9%)	1,015 (3.4%)

### Distribution of fracture costs

**Figure 2:**
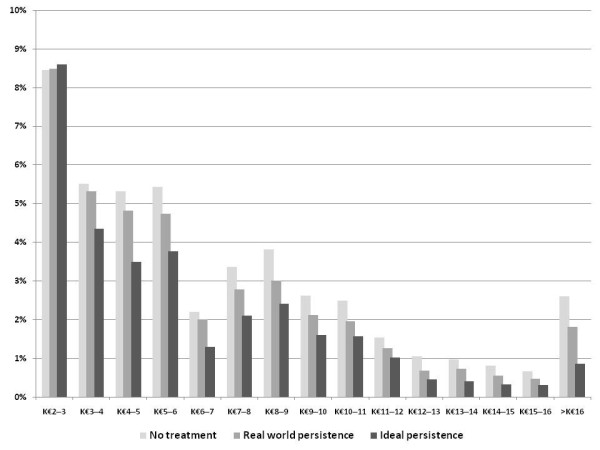
**Distribution of 10-year fracture cost management**.

Within fractured women population, mean costs of 10-year management of fracture were significantly different between the three alternatives with €7,239 (± €4,783), €6,711 (± €4,410) and €6,134 (± €3,945) in the no-treatment, the real-world and ideal persistence alternatives, respectively (p < 0.0001). The distribution graph showed left-shifted medians and a spread of costs on the right side (Figure 2). Compared to no-treatment and real-world persistence alternatives, all proportions of cost ranges over €3,000 decreased in the ideal persistence alternative.

### Cost-effectiveness analysis

Cost-effectiveness ratio for real-world treatment persistence compared with no-treatment alternative was found dominant and as well, alternative of ideal persistence dominated the former (Table 4). One-way sensitivity analysis of discount rate did not affect direction of those results. However, when bisphosphonates current branded prices were considered, the cost per fractured women saved and premature death avoided were respectively €309 and €2,251 for ideal persistence alternative compared to real-world one.

**Table 4:** Cost-effectiveness of bisphosphonates for real-world and ideal persistence alternatives

Alternatives		No-treatment	Real-world persistence	Ideal persistence
**Costs (Discounted)**	Average per patients	€3,402	€3,110	€2,833
	Incremental	--	-€293*	-€277**

***Costs (Undiscounted)***	*Average per patients*	*4,428 €*	*€3,979*	*€3,529*
	*Incremental*	*--*	-€449*	-€450**

**Fractured women**	Proportion	0.671	0.600	0.491
	Incremental	--	-0.071^†^	-0.109^††^
**ICER**	Discounted	--	(Dominated) €4,110	(Dominated) €2,535
	*Undiscounted*	*--*	*(Dominated)* *€6,310*	*(Dominated)* *€4,114*

**Premature deaths**	Proportion	0.061	0.049	0.034
	Incremental	--	-0,012^†^	-0,015^††^
**ICER**	Discounted	--	(Dominated) €25,073	(Dominated) €18,442
	*Undiscounted*	*--*	*(Dominated)* *€38,489*	*(Dominated)* *€29,932*

* *μc*_Real-world persistence_--*μc*_No treatment_; ** *μc*_Ideal persistence_--*μc*_Real-world persistence_

^† ^*μE*_Real-world persistence_--*μE*_No treatment_; ^†† ^*μE*_Ideal persistence_--*μE*_Real-world persistence_

### Cost of non persistence

**Figure 3:**
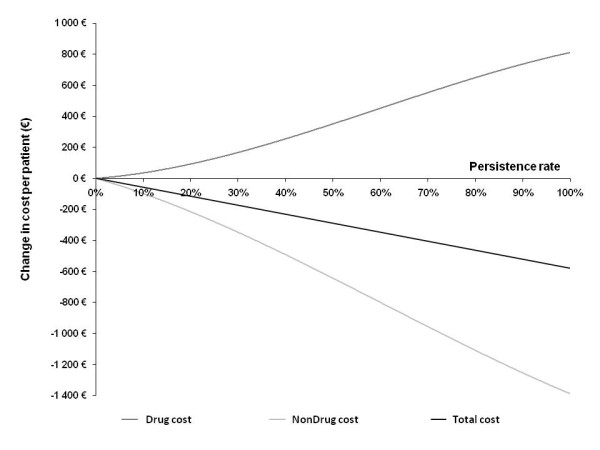
**Change in drug and nondrug costs in function of persistence**.

Ten-year changes in costs of management of fracture (i.e. nondrug costs) and drug costs in function of persistence are illustrated in Figure 3. Total cost changes, obtained as sum of drug and nondrug costs, represented savings from the beginning of a pseudo-linear curve to the level of ideal persistence (i.e. 100%). Each ten percentage points of persistence gain amounted to €58 per patient. Hence, the cost of non-persistence as the difference between total costs of ideal persistence alternative and real-world persistence alternative achieved €270 per patient over the 10-years period. When extrapolated, this result allowed an estimation of the global cost of non-persistence in France to €30.5 million per year.

## Discussion

In a context of generic prices, the hypothetical alternative of ideal persistence with oral bisphosphonates was shown economically dominant on the real-world persistence. Moreover, the savings came to €58 per patient for each ten percentage points of persistence gain. The extrapolated global cost of non-persistence in France was also estimated to around €30.5 million per year.

Those results demonstrate the extent of the economic impact of non-persistence with oral bisphosphonates and as a corollary, the potential lever of improving this parameter. Although achieving an ideal persistence for the whole osteoporotic women seems illusive, few strategies have already demonstrated relevant efficacy in enhancing persistence. The first of all was the development of intermittent administrations allowed by pharmacokinetics specificity of bisphosphonates [37]. Alendronate and risedronate have been formerly available with oral daily regimen and then both developed with oral weekly regimen. Although persistence with bisphosphonate treatments has been improved with those weekly formulations, it was still shown to be suboptimal in many western countries [12]. The search for adherence optimization leads other bisphosphonates providers to develop further the concept of intermittent dosing regimen. In 2006, ibandronate was the first within the bisphosphonate class being proposed with a monthly regimen to post-menopausal women, recently followed by risedronate [38]. Topical data confirmed this strategy with a proportion of persistent patients achieving 17 percentage points higher with monthly regimen compared to weekly users after 1-year [39]. Seeing the quick and almost full switch of bisphosphonates with daily to weekly dosing regimen [7], it is plausible that monthly dosing regimen would become one day the standard of oral bisphosphonates dosing frequency. However, due to a lack of proven efficacy on hip site, French authorities recently decided that ibandronate would not be reimbursed anymore. Intravenous bisphosphonates are now available with yearly or quarterly dosing regimen with, in essence, a sustained persistence. Those intravenous bisphosphonates may be particularly useful to treat patients with a high risk of non-persistence who could be simply identified by validated tools, such as the recent ADEOS-12 items questionnaire [40]. Institutionalized patients may also easily benefit from those specific administration routes. However, experience has shown higher patient's preference and acceptability for oral administration than for hospital infusion, in the case of chemotherapy notably [41].

In the past twenty years, the global cost of non-adherence with medications was successively estimated in US at 20 million lost work days and $1.5 billion in lost earnings annually in 1990 [42], at $100 billion annually in 1997 [43] and finally more than $300 billion annually in 2004 [44]. Outdated but frequently cited in literature introductions, those macro-economic data suffered from numbers of methodological biases and have to be used with caution according to the author of the latter. In general, when taking the drug costs into account, it is not clear whether improving adherence with chronic medications reduces or increases costs and treatment-specific or target-population-specific studies are needed. Very few surveys identified increased healthcare costs due to medication non-adherence against offsets from reduced drug intake. In Parkinson's disease and epilepsy, recent database analyses showed a large net positive effect of non-adherence on total annual health care costs remained despite savings in pharmacy costs [45,46].

Four previous studies used mathematical simulation models to calculate the lost benefits associated with real-world non-adherence in very different populations: renal transplanted subjects [47], HIV [48], hypertensive [49] and schizophrenic patients [50]. Unlike in our present results, ideal adherence with renal transplant, HIV and hypertension medications was not found as a dominant strategy, but cost-effective with €35,000 per Quality-Adjusted-Life-Year (QALY), $29,400 per QALY and $22,100 per life-year saved, respectively. In those cases, results were equivalent of an estimation of the cost-effectiveness of interventions to improve adherence to levels observed in clinical trials. The latter study predicts that increases in compliance with anti-psychotics may lead to considerable cost savings and health improvements. Hence, each percentage point of compliance gain was predicted cost saving over 5 years of around €650 and a QALY gain of 0.004. According to our study, spending up to €270 per osteoporotic patients in an effective adherence-enhancing intervention would result in economic neutrality. Over this amount a cost-effectiveness trade-off would be needed. In comparison, adherence-enhancing interventions to asthmatic and lipid patients were evaluated as $32 [51] and $182 [52] offsets, respectively.

Model type, structure, validation have been already fully discussed in a previous publication as well as its stability [18]. Estimation of fracture costs were based from the analysis of an exhaustive National database and, regarding hip and wrist fractures, were consistent with previous ones [35]. However, it was not possible to identified vertebral fractures caused by trauma or malignant tumours from those effectively due to osteoporosis and, it was assumed that management and also hospitalization costs were similar, whatever the original causes of fractures. Several factors may contribute to a probable underestimation of the actual costs in this model. The majority of the vertebral fractures do not require hospitalization and were assumed as asymptomatic with no associated cost. However, many of those fractures probably lead to other ambulatory resource utilizations (e.g. back pain treatment). As well, due to the lack of data, our estimations of fractures costs did not take into account the patient transport or indirect costs.

Our clinical assumptions and input data were also clearly stated and justified as required by guidelines for pharmacoeconomic research. Cost-effectiveness results were expressed with non conventional ICERs compared to those mostly used in the literature, especially QALYs. The latter are actually not recommended by French economic evaluation guidelines for public decision making [53]. Furthermore, outcomes chosen in this model (i.e. proportion of fractured women and premature deaths) could be considered as more clinically meaningful than aggregate ones.

## Conclusion

Based on the results of the model, improving persistence with oral bisphosphonates was shown economically dominant on the real-world persistence. When extrapolated to the whole of France, savings could rise to over €30 million per year, with a substantial transfer from hospital to pharmacy budgets. Pharmacoeconomic environment in the field of osteoporosis offers new opportunity to optimize patients' management on the condition that cost-effective adherence-enhancing interventions be identified and carried out.

## Competing interests

GdP has no competing interests. FEC has been employed at Laboratoire GlaxoSmithKline (GSK) who markets anti-osteoporosis treatments (ibandronate and denosumab). The study was funded, in part, by "Association Nationale de la Recherche et de la Technologie", with the support of GSK.

## Authors' contributions

FEC and GdP carried out the whole research process. FEC designed the model framework, performed the cost analyses and drafted the manuscript. GdP advised on the study design, contributed to the interpretation of the results and critically revised the manuscript. Both authors read and approved the final manuscript.

## Pre-publication history

The pre-publication history for this paper can be accessed here:

http://www.biomedcentral.com/1472-6963/11/151/prepub
